# GPs' perspectives of type 2 diabetes patients' adherence to treatment: A qualitative analysis of barriers and solutions

**DOI:** 10.1186/1471-2296-6-20

**Published:** 2005-05-12

**Authors:** Johan Wens, Etienne Vermeire, Paul Van Royen, Bernard Sabbe, Joke Denekens

**Affiliations:** 1 Department of Family Practice, University of Antwerp – Faculty of Medicine, Universiteitsplein 1, 2610 Wilrijk (Antwerpen), Belgium; 2 Department of Psychiatry, University of Antwerp – Faculty of Medicine, Universiteitsplein 1, 2610 Wilrijk (Antwerpen), Belgium

## Abstract

**Background:**

The problem of poor compliance/adherence to prescribed treatments is very complex. Health professionals are rarely being asked how they handle the patient's (poor) therapy compliance/adherence. In this study, we examine explicitly the physicians' expectations of their diabetes patients' compliance/adherence. The objectives of our study were: (1) to elicit problems physicians encounter with type 2 diabetes patients' adherence to treatment recommendations; (2) to search for solutions and (3) to discover escape mechanisms in case of frustration.

**Methods:**

In a descriptive qualitative study, we explored the thoughts and feelings of general practitioners (GPs) on patients' compliance/adherence. Forty interested GPs could be recruited for focus group participation. Five open ended questions were derived on the one hand from a similar qualitative study on compliance/adherence in patients living with type 2 diabetes and on the other hand from the results of a comprehensive review of recent literature on compliance/adherence. A well-trained diabetes nurse guided the GPs through the focus group sessions while an observer was attentive for non-verbal communication and interactions between participants. All focus groups were audio taped and transcribed for content analysis. Two researchers independently performed the initial coding. A first draft with results was sent to all participants for agreement on content and comprehensiveness.

**Results:**

General practitioners experience problems with the patient's deficient knowledge and the fact they minimize the consequences of having and living with diabetes. It appears that great confidence in modern medical science does not stimulate many changes in life style. Doctors tend to be frustrated because their patients do not achieve the common Evidence Based Medicine (EBM) objectives, i.e. on health behavior and metabolic control. Relevant solutions, derived from qualitative studies, for better compliance/adherence seem to be communication, tailored and shared care. GPs felt that a structured consultation and follow-up in a multidisciplinary team might help to increase compliance/adherence. It was recognized that the GP's efforts do not always meet the patients' health expectations. This initiates GPs' frustration and leads to a paternalistic attitude, which may induce anxiety in the patient. GPs often assume that the best methods to increase compliance/adherence are shocking the patients, putting pressure on them and threatening to refer them to hospital.

**Conclusion:**

GPs identified a number of problems with compliance/adherence and suggested solutions to improve it. GPs need communication skills to cope with patients' expectations and evidence based goals in a tailored approach to diabetes care.

## Background

Diabetes mellitus type 2 is an important and increasing health problem. In Belgium, the incidence is 231 new cases per 100.000 inhabitants per year [1]. It is frequently not diagnosed until complications appear, and approximately one-third of all people with diabetes may remain undiagnosed. The estimated prevalence of diabetes among adults was 7.4 % in 1995; this is expected to rise to about 9 % in 2025 [2]. To date there is strong evidence that vigorous treatment of diabetes type 2 can decrease the morbidity and mortality of the disease by decreasing its chronic complications [3-6]. However, poor patient compliance/adherence to these treatment recommendations can reduce therapeutic effects.

Earlier research on compliance/adherence showed that neither the features of a disease, nor the referral process, nor the clinical setting nor the therapeutic regimen seem to influence compliance/adherence [7]. Because of the difficulties in measuring, no estimate of compliance/adherence or non-compliance/non-adherence can be generalized. Poor compliance/adherence is to be expected in 30–50 % of all patients, irrespective of disease, prognosis or setting [8-10]. Today, more then 200 different doctor- patient- and encounter-related variables have been studied but none of them is consistently related to compliance or fully predictive. Especially in quantitative studies, little attention has been paid to patients' ideas about medicines and compliance/adherence. However, from qualitative research we know that the most salient influences on compliance/adherence are patients' own beliefs about medications and about medicine in general [7]. Their own knowledge, ideas and experiences, as well as those of family members and friends, have also been shown to correlate with compliance [11].

In order to understand and predict compliance/adherence, the patients' attitude towards disease has been studied since more then twenty years by means of the health belief model [12,13]. Today, new concepts of patient involvement, participation and real partnership are introduced [14]. Therapeutic interactions with patients should not longer be viewed simply as opportunities to reinforce instructions around treatment: rather, they should be seen as a space where the expertise of patients and health professionals can be pooled to arrive at mutually agreed goals [15]. In primary care, patients strongly want a patient centered approach, with communication, partnership and health promotion [16]. Evidence is increasing that involving patients more in consultations can increase compliance/adherence to treatment.

Patients and caregivers interpret signs and signals in a different way [17]. These differences in perspective are not inherently problematic. They frequently become so when patients do not meet the goals and expectations of their health care providers. In caregiver's point of view there is an expectation that when they, as authoritative experts, make recommendations, the patient only has the obligation to carry them out [18]. Furthermore, caregivers are increasingly under pressure from health policy makers or managed care organizations to reduce the costs of complications by rigorously complying with treatment guidelines.

Physicians estimate their patients' compliance/adherence to medications, and base decisions about treatment on these estimates. Misjudgment of patient compliance/adherence can have adverse consequences, including withholding of therapy or unnecessary changes in therapy. A review of the literature demonstrates that physicians are often inaccurate in estimating patient compliance/adherence with antiretroviral therapy [19]. In the field of diabetes, medical anthropologists evaluated an analytical framework for contrasting patient and provider goals and strategies [20]. This framework was designed to allow examination of how a chronic illness fits into both clinical and life-world contexts. By comparing patients' and providers' goals, strategies and evaluation criteria, a better understanding could be developed of how recommended behavioral changes are understood and applied differentially by each group. Using this approach, certain shared outward semantic similarities (i.e. "control") were found between patients' and providers' perspectives that obscured the presence of deeper differences between those perspectives. The differences had important implications for the long-term enactment of diabetes self-care regimes.

The clinical literature on "non-compliance/non-adherence" tends to problematize only the patient's perspective, treating the provider's perspective as an uncontroversial point of departure. Equal consideration is seldom given to the role of provider perspectives and the broader institutional and economic contexts of illness management [21]. Our work on compliance/adherence focusing on the providers' perspective, illustrates the difficulties Belgian GPs experience in trying to help people with diabetes type 2 being adherent to treatment plans.

## Methods

This descriptive qualitative study explores the thoughts and feelings of GPs on patients' compliance/adherence. In five focus groups (FG), GPs were asked how they think their type 2 diabetes patients adhere to life style and dietary advices, medicine taking and management of the disease. We sounded out how GPs cope with conflicts concerning these topics. In our analysis, we tried to explore the barriers to compliance/adherence and possible solutions, GPs experience in diabetes management. We also explored some coping mechanisms they use to handle the conflict arising when patients do not heed their advices.

The focus group technique used was based on a standardized procedure, described in earlier publications [22-24].

After approval of the different local societies for primary care and of the ethics committee of the study centre, all GP members (n = 173) working in the surrounding municipalities of the study centre received a letter with some general information on our project. Afterwards, physicians were phoned to assess their interests in participating and subscription in one of the discussion groups planned. Forty interested physicians could be recruited and five focus group sessions were organized. In compiling the different groups, no distinctions were made on age, gender or number of years in practice in the hope of maximizing interaction and outcome. Interested physicians could make their choice between an afternoon and an evening session. They also could choose between a venue on the university campus or not. If after the five focus groups content saturation was not achieved, additional focus groups were planned.

A previous qualitative study on adherence to treatment recommendations in patients living with diabetes [25] revealed five different themes on the problem of compliance/adherence. Together with the results of a comprehensive review of recent literature on adherence [26], these themes were used to generate five open-ended core questions (table 1) exploring the ideas and thoughts of the participant GPs. The precise formulation of these questions was discussed thoroughly by the research team until mutual agreement and piloted in a first focus group.

**Table 1:** Focus group questions

Research questions:

FG Question 1 Introduction

• Do you as a physician have some insight in how your diabetes patients think about their illness and the treatment necessary to them?

FG Question 2 Health beliefs and advice giving

• In your treatment, you give many advices as on diet, walking, smoking cessation etc. Perhaps there is a good intention for it. However, can you tell us what you think your patients do with these advices?
• It might be possible that physicians do not understand enough the health beliefs of their patients when they give their advices. What do you think about this?

FG Question 3 Health beliefs on drug intake

• In addition, when you prescribe your medicines you have some expectations about your patients. What is your experience as to patients fulfill these expectations or not?
• Probably, patients have some alternative expectations. Do you notice that?
• It might be possible that physicians do not understand enough the health beliefs of their patients when they prescribe their medicines. What do you think about this?

FG Question 4 Health beliefs on follow up

• The guideline level on follow up of diabetes patients is not easy at all. Many examinations have to be performed; appointments have to be made to monitor the continuous changing risk profile of the diabetes patient. What do you think the diabetes patient expects from all this?
• It might be possible that physicians do not understand enough the health beliefs of their patients when they plan their follow up. What do you think about this?

FG Question 5 Coping with conflicts

• Sometimes, you certainly may not reach common grounds with your patients. How do you express these conflicts?
• How do you handle them?
• What solutions can you suggest?

A diabetes nurse, well-trained and experienced in conduction focus group discussions, moderated all focus groups, guaranteeing a standardized procedure. She observed that the level of involvement was as non-directive as possible, but meanwhile trying to collect as much data as possible, ensuring that the desired set of topics was covered and encouraging the participation of everyone [27,28].

An observer (JW) gathered information on the non-verbal communication and on the interaction between participants. In this way, special attention could be given in the analysis, to those pronouncements and text fragments where verbal and non-verbal consensus existed. The duration of the discussions was limited to two hours.

At the end of every focus group, there was a debriefing between moderator and observer to discuss the most important themes and possible differences with other focus groups. Since participants of FG 5 added no new information anymore, content saturation was supposed to be reached.

The first focus group served as a pilot. Since no indistinctness with regard to the questioning or other procedures were allocated, and since the results were very similar to the other focus group interviews we decided to keep these first results in the analyzing process. In no focus group, any striking nuisance cases were present.

All focus groups were audio taped and transcribed for content analysis.

Two researchers (JW and EV) independently performed the initial coding. In the case of disagreement, a solution was found by clarifying individually the meaning of a code and discussing until mutual consent was reached. We defined the different themes and performed cross-referencing within the different coding categories, and by text word search in the document or selected sections of it. In doing so, we clarified ideas, discovered further themes, searched for patterns and explored participants' explanatory models.

A first draft with results was sent to all participants for agreement on content and comprehensiveness (response rate 72.5 %). This participant checking leaded to only a few interpretative comments. We added or corrected most of them in the draft after discussion and agreement with the moderator.

## Results

Of the 173 GPs contacted, 61 agreed to participate (35.3 %), and 40 GPs (23.1 %) attended the focus groups. Physician and practice characteristics are summarized in table 2.

**Table 2:** physician and practice characteristics

	FG 1	FG 2	FG 3	FG 4	FG 5	Total
N	10	7	9	8	6	40
						
♂ / ♀	7 / 3	4 / 3	6 / 3	4 / 4	5 / 1	26 / 14
						
Mean age (S.D.)	44.8 (9.8)	47.9 (11.8)	48.6 (9.0)	38.4 (5.8)	47.5 (15.3)	45.3 (10.5)
						
mean years in practice (S.D.)	18.1 (9.9)	21.9 (11.4)	20.5 (8.4)	12.5 (5.9)	19.2 (16.2)	18.4 (10.3)
						
Solo (%)	70	71	67	88	67	73

N = number of participants

S.D. = standard deviation

Content analysis of the text fragments resulted in primary codes, which after an inductive interpretation and categorization process could be structured in three themes (table 3): barriers to patient's compliance/adherence as experienced by the GPs; solutions as used by the GPs to increase their patients' compliance/adherence and coping mechanisms used by the GPs.

**Table 3:** Themes, categories and codes.

Theme	code
Barriers to adherence	
Patient	Categorical approach
	Social isolation
	Deficient knowledge of diabetes
	Minimising of the disease
	Opposition to change
	'Modern' medicine
	
Physician	Choosing the easiest way
	Supposed lack of respect
	
Model of health care	No repayment for self-monitoring
	Lack of multidisciplinary support

Solutions	
Communication	Evaluate knowledge
	Give information
	Repeat
	Control how it can be achieved
	In the patient's language
	
Work in a structured way	Make appointments
	Standardized educational packages
	Structured file administration
	
Shared care	Multidisciplinary teams
	Identical messages

Coping mechanisms	
Physicians	Directing /paternalistic attitude
	Induction of guilt and fright
	
Patients	Discussed decision-making

### Barriers to compliance/adherence

With regard to compliance/adherence, GPs recognized two kinds of patients: "motivated" patients and "not-to-be-motivated" patients. The "good" patients were very motivated, they did their best and they almost meticulously followed their regimens. The "bad" patients were hardly to be motivated, they thought they have everything under control but they were neglectful which was frustrating for the GP.

M. *I think you can hardly speak about "the" diabetes patients; at least this is what I see in my surgery. You have people who are very conscious of their illness, they really take care and on the other hand, there are the ones who neglect everything. I bet everybody here knows these kinds of patients *[general agreement]. *So, all these different people have a different attitude towards their disease and their treatment in general*.

Though they know it is an important challenge and duty, GPs were convinced that it is very difficult to motivate their patients to adhere to treatment regimens. They considered barriers for compliance/adherence on three different levels: the patient, the GP and the care model in which the treatment is offered.

#### The patient

At the patient's level, their GPs identified important barriers such as social isolation and deficient knowledge of diabetes. They also remarked that diabetes patients tend to minimize their disease. This really is in contrast with the GP's objectives.

F. *I believe patients often do underestimate diabetes *[general agreement of the group]. *The patients think it is an infirmity of old age, an age disease. Consequently, they are not motivated to follow strictly their diet and therapy. That is my conclusion after 30 years of practice*.

GPs acknowledged the kind of problems patients have with the treatment regimen and realized that a lifestyle change is not that simple. Yet, they felt the patient's opposition by lots of excuses for preservation of non-healthy habits.

On the other hand, some patients have learned to rely on modern medicine that offers medication for every complaint. To take that medicine is easier and fits better in our way of life than to part with dietary habits linked to the patient's cultural environment.

P. *If you feel ill, then you take a pill... anyway, there are drugs for all complaints*.

#### The general practitioner

GPs quickly begin to feel powerless once they experience that patients do not attain the EBM treatment goals. This has a negative impact on further promotion of compliance/adherence to proposed regimens. GPs cherish high expectations concerning metabolic results in their diabetes patients, but do not succeed in communicating this to their patients. Few GPs liked to repeat time and again the same messages, they didn't like to "nag" or "tease" as it is mentioned in one of the focus groups, as this simply leads to frustration.

W. *Of course we must ask ourselves if we are doing the right thing. Is it really so bad to give a pill when the patient is satisfied, when the sugar levels are OK and the HbA1c is good, but when the patient doesn't strictly follow his diet. What do we have to do then?*

B. *When the patient persists in neglecting his treatment, I give up*...

#### The care model

With regard to diabetes care, the Belgian health care system uses a convention system in which intramural (secondary care) centers arrange with the National Institute for Disease and Disability Insurance (RIZIV/INAMI) to refund well-defined diabetes care to specific patients. To be considered for this care, at least two insulin injections a day are required. On this condition, patients have a right to free dietary advice and diabetes education and free (but limited) materials for self-monitoring.

For all other diabetes patients, mostly treated in primary care, currently there is no reimbursement for education or self-monitoring of blood glucose. The GP felt that his good relationship with the patient is threatened by this inequality, because others can offer their patients advantages that they as GPs cannot give. In the GPs opinion, this interference in the relationship of trust could hinder the compliance/adherence. The superior position of the free accessible "specialist", who may have another message for the patient, also gave many GPs a bad feeling.

P. *Our experience points out that some of these centers take over the care of patients and that we never see those patients again. We still get reports and results of blood tests on a regular base but we do not see the patient any more*...

Poor co-operation between intra-mural diabetes centers and the GPs divides the care process between different partners ignoring each other's efforts and responsibilities. Moreover, in the system of fee-for-service the GP often has to go along with the expectations of the patient in order to keep him in his surgery and to protect his income. This made the physician thinks his patients do not appreciate his specific expertise and skills, which again may result in frustration and even anger.

G. *Get angry *... [sigh], *but afterwards I feel so frustrated. Get angry with somebody to whom you could have explained in an adult way, "this is becoming impossible", "I do it this way, that's my routine and we'll try stick to it." When I get angry, it is on my mind for the rest of the day*...

### Solutions

Solutions were identified in three categories. Core elements hereby are: communication, tailored care and shared care.

### Communication

GPs realized they have opportunities for communication with the patient. They knew it is wise to check first what the patient already knows, in order to give further relevant information, taking into account the patient's ability to assimilate the messages. GPs should repeat information, check the understanding; explore the patients' own thoughts about and the willingness to apply them.

Doing so, GPs should be careful not to overload patients with information and not to keep "harping on" about health advice that does not (yet) interest the patient.

A. *I wonder if you haven't to be careful with those explanations. You see, if each time you give a flow of information, you'll make the patient sick with it ... He'll get demotivated, especially when, after a blood sample, showing a bad result while the patient himself thinks he has done his best. You must not exaggerate in trying to give advice*.

### Tailored care

GPs considered that the way in which a consultation is carried out could also stimulate compliance/adherence. Important factors seen as able to increase compliance/adherence were scheduled consultations and teamwork. It should give the patient a better notion of "care giving" when a structured diabetes consultation model is adopted. This, together with consultation hours by appointment give patients a feeling of more time being spent and of more experience being applied on his/her behalf. GPs thought that standardized education tools and a structured diabetes file could improve compliance/adherence compared with an "ad hoc" consultation in which the GP only tends to go along with patients' complaints and questions.

B. *I think it's partly our fault. I have the impression I cannot follow up so easily and I feel you do it in a more structured way. It all depends on that. So "How does one handle it, how can one work in a structured way on his own?" If you do so, the patient follows. In my case, it is not very structured so far and I don't follow up very well. Therefore, my patients don't do it either. I think that can make a difference when you handle it professionally and structured*.

### Shared care

GPs considered that working in a multi disciplinary team might encourage better compliance/adherence. They believed that dieticians could give more adequate and more varied food advice than they could themselves. When they succeeded in referring the patients to the dietician, the majority of patients were very satisfied, but the first step remained difficult.

T. *I find out that it's not sufficient when we give advice. That's why I usually send the patients, as soon as possible, to the diabetic dietician who gives them explanations. When 2 or 3 people co-operate, it works better. The patients follow up more strictly then when a doctor works on his own. A doctor might also give a leaflet about diabetes, the patients read it but next time, they have forgotten everything about it. They put it somewhere, stuffed away in a drawer. When a dietician sees the patient, he asks what he eats. All that practical information makes a stronger impression than the physician's advice. Besides, I don't have enough time to give such detailed explanations*.

### Coping mechanisms

GPs often become directing and paternalistic in order to cope more easily with the above-mentioned barriers. This attitude may induce guilt and anxiety in the patients. Physicians assumed that shocking the patients, pressuring and threatening to send them to the hospital were often the only effective methods to improve compliance/adherence.

W. *It all depends on the characters, you see. You have people who never feel responsible; they always put the blame on somebody else, despite all your explanations. If something bad happens, they'll tell you you've never explained enough, never emphasized enough or never frightened them enough. Because, you say sometimes "You should not frighten them." However, for some people the only option is to make them afraid. Otherwise, they won't do it and then they blame you for not doing it*...

In this way, GPs tighten a net around the patient, restricting his/her freedom of movement. An indication of this restriction can be found in the explicit request for a more tailored care system. In emphasizing this, doctors may minimize their own responsibility and try to shift the blame to the liberal marginal conditions in which they are functioning.

Physicians also considered that patients take refuge behind different reasons in an argued decision, such as cultural motives (for example dietary tradition), financial problems (lack of reimbursement) and strict personal reasons to avoid compliance/adherence. They viewed the motives of patients as deep-rooted and difficult to modify.

## Discussion

Many patients for whom diabetes medication is prescribed are poor compliers with treatment, including both oral medication and insulin [29]. In these exploratory focus groups, we discover barriers and solutions for optimal compliance/adherence in people living with diabetes type 2, seen by Belgian GPs.

From the dynamic discussions in the focus groups, we may conclude that this subject was of substantial concern to GPs. There was a constructive mutual interaction and good co-operation in all five focus groups. Open discussions went along with a positive willingness to participate in the debate.

From the GPs' point of view, an inventory could be made of the different barriers and solutions that matter for compliance/adherence of their diabetes patients. This inventory is far from complete. Besides, it was not the intention of this study to make out an exhaustive list of all kinds of compliance/adherence barriers. In this article, we confine ourselves to the viewpoint on compliance/adherence by diabetes patients, as experienced by the GP.

We learn that GPs take a shot at the patients, complaining their deficient knowledge, the fact they minimize diabetes related problems and their blind confidence in medicine. GPs also blame the health care system in which they feel as inferior doctors in comparison with specialists, widely accessible for patients. Taken together, these externalizing factors seem to lead to a profound defeatism. Where the GP tries to give "good" diabetes care by strictly promoting evidence-based diabetes objectives, the patients' "failing" frustrates him. Besides, they recognize the lack of effective communication tools for making the patient a real partner in their decisions.

Probably, GPs feel inclined to allocate the extent of someone's responsibilities too fast. Perhaps, they take too much personal responsibility and focus too much on evidence based medical treatment options wherefore they are real experts and responsible for. A failing in reaching these treatment goals then is a failing of the doctor-expert himself who is doing the best he can. Lifelong guidance of chronic ill patients as is the case in diabetes, asks for shared responsibilities where going about with the disease (behavioral changes, compliance/adherence attitudes, ...) is the patient's responsibility. New essential skills and attributes for a health care provider to promote behavioral change and risk reduction are skills for assessing readiness for behavioral change, relationship building skills, and skills in considering the patient's attitudes and beliefs about the disease or treatment involved [30]. Motivational interviewing, characterized by its empathic non-confrontive style, assists movement through the stages of change to the "action" stage where engaging in change behavior begins. It is designed to assist clients (patients) in exploring and resolving ambivalence to increase motivation for change. Physicians can use the model to intervene successfully at all different levels of behavioral change, where patients are helped by the model to take responsibility for changing habits [31].

Lacking these skills of motivational counseling and shared decision making, the best method to promote patient's compliance/adherence is assumed to be using shock tactics. Identical conclusions were found in a Polish study were doctors, when confronted with non-compliance, also applied certain tactics of doubtful effectiveness which might worsen the patient's emotional state or harm his as a person. Valuable techniques of proven efficacy were not used [32].

Tailored and shared care is considered as a possible solution for better compliance/adherence of diabetes patients. From GPs point of view, this long for teamwork fits with emerging international evidence stating that structured [33] and patient oriented [34] care result in better outcomes. This new model of care fits quite well with our medical paradigms, but might be too much adaptedto the caregivers, without taking into account the views and priorities of (chronic) patients. It remains to be seen if structured care can also deliver a better long term care of patients with chronic illnesses. Don't we forget the patient in organizing shared care?

The cornerstones of health care to support active patient participation are to guarantee the continuity of care, to integrate education in health care and to encourage the patient's attendance [35]. An increased patient satisfaction may be contributing to improved clinical outcomes [36]. However, the relation between patients' satisfaction and GPs patient-centered behaviors remains unclear [37]. There is also limited and mixed evidence on the effects of patient centered interventions on patient health care behaviors or health status; or on whether these interventions might be applicable to providers other than physicians as diabetes-specialized nurses [38]. Professionals committed to achieving the benefits of patient centered consulting should take care not to lose the focus on disease while paying attention to the unique experience of illness of each patient [39]. Enhancing patient-provider agreement on both overall treatment goals and specific strategies to meet these goals may lead to improved patient outcomes [40].

Until now, little research has being done on the relationship between health care provider and compliance/adherence. For HIV patients, better treatment adherence is reported in those patients who perceived themselves to be more engaged with their health care provider [41]. However, since self-reported measures were used, this finding should be viewed with caution. Also in antidepressant treatments, the physician's attitude about the medication is a key factor for improving compliance [42].

A Canadian study on the role of patient, physician and system factors in the management of type 2 diabetes patients [43] shows very striking similarities with our study on compliance/adherence. Canadian GPs also believe that early educational interventions for patients with diabetes resulted in better outcomes. They also described the diabetes educational centers as a valuable resource and stressed the importance of referring the patient soon after diagnosis. In Canada, time and physician remuneration were identified as the main health system barriers to optimal diabetes management.

Qualitative research is often deemed to have lower reliability compared with experimental research. Yet, it has strong face validity, especially when it includes an observational component that enables the researcher to compare oral statements with actual practice [44]. In all our focus groups, all participating GPs spoke very spontaneous in an open atmosphere without any verbal aggression. However, in answering the questions, there was a clear sound of frustration in all five focus groups. Probably, that is a reason why some of the quotes sound rather punitive and blaming of patients. We never had the feeling that GPs were "set up" to respond in such a way. They react defensively because they feel powerless. They are afraid of decreasing income and standing by loosing patients in a fee-for-service payment with free entrance to all levels of care.

At least some limitations to our study have to be considered.

First, the topic of interest. Research in such a complicated matter as compliance/adherence is extremely difficult and always fragmentary, especially given the lack of a specific model or general theory. Different authors use "compliance", "concordance" and "adherence" haphazardly, complicating a comprehensive study of the literature [26]. Additional, for instance in Dutch, the three different meanings of compliance, concordance and adherence are interpreted only by one word. Though we presume in this context that "compliance" (doing what is told to be done) is the best translation, we prefer to use the combined term "compliance/adherence".

Second, the sampling. The participating GPs in this study were all volunteering and interested in a discussion on "problems diabetes patients might have to follow treatment recommendations", as mentioned in the introduction letter. Perhaps, volunteering GPs are more willing to discuss these difficult problems in the patient provider interaction. Since our sample fits well with age, gender and practice organization (table 2) of the Flemish GP population, we assume having described a maximal variation of answers. Besides, the main goal of our study was not to generate conclusions that could be generalized, but to explore and gain a deeper understanding of the GPs perspective of patient compliance/adherence. This limitation should also be taken into account by interpreting the results and before transferring the conclusions to other contexts. It could be argued that selecting GPs in different focus groups according to gender or experience could have been a more appropriate approach. The number of years in practice might be influence frustration. However, the provoked diversity of the group was meant to elicit a rich dynamic interaction in the conducted conversation.

Third, the analysis. While looking for barriers and solutions in patients' adherence GPs' frustration is an important issue in our results. GPs feel as if the patient does not consider them capable to treat their illness. However, a recent and extensive health inquiry in Belgium confirms the people's confidence in general medicine [45]. More, patients' own choice of a primary care physician seems to be associated with better adherence to their treatment regimes [46]. This relationship even remained significant after controlling for possible confounding factors as physician gender, patient gender or length of relationship. Perhaps GPs underestimate their potential value and the effect they can achieve with their patients. Rather than reflecting on themselves and making themselves familiar with newer communication skills, GPs shift the blame of failing on an inappropriate system or other extrinsic factors (e.g. cultural and social problems).

Patients, especially those with chronic illness, make decisions about treatments that fit with their own beliefs and personal circumstances. They may have clear reasons, narrowly related to their health beliefs, for adhering or not adhering to treatment recommendations.

The shared decision making process is rather complex. A joint patient-provider perspective is needed. Health care professionals should assess patients' reasons and beliefs in order to achieve agreement on attainable treatment goals. They need to shift the emphasis away from attempting to directing patients into take the medication they prescribe, towards learning how they can contribute to the decisions that patients currently make about their medications [47].

Adherence to treatment is therefore the shared responsibility of patients and GPs. In complex treatment plans, as is the case in diabetes, a possible way of helping people being adherent perhaps is helping them making more explicit their own attainable priorities. Exploring thepatient's expectations towards the disease and its treatment and translating these individual expectations to realizable and realistic objectives for the patient and in concert with the patient, is an important communication task for the GP. In fact, it is patients who should be the primary actors in medical decision-making, and the health professionals should adopt a supportive role. This joint patient-provider interaction could be a real contribution to a more positive approach on diabetes care. It will help patients to set their own desirable goals and give the GPs' consult again more value and depth. . In essence, then, compliance is an elusive, flexible goal.

## Conclusion

The patient's own expectations with regard to illness and health not always correspond to the objectives and expectations of the physician's treatment proposals. The motivation of the physician to achieve a good result may be in conflict with the patient's own motivation to lead his own life. GPs seem to be in need of communication skills to integrate the various expectations of physicians and patients regarding diabetes care.

Our findings suggest a necessary shift to a model of patient-provider-partnership with mutual agreement on shared decisions. A closer relationship wherein the patient is more engaged to his treating GP possibly could reduce frustration.

Shared care, referred by these GPs as a possible solution for better compliance/adherence, only can fulfill these purpose if, from the beginning, the patients' perspectives and concerns are not forgotten.

## Competing interests

The author(s) declare that they have no competing interests.

## Authors' contributions

JW carried out the design of the study; he is responsible for the phrasing of the focus group questioning, compounding the codebook and analysis of all transcripts. He drafted and finalised the manuscript; EV participated in discussing the concept of the project, question phrasing and the coding process. He made a major contribution to rewriting all draft versions of the manuscript; PVR also participated in the definitive phrasing of the questions and critically revised the manuscript as did BS and JD. All authors read and approved the final manuscript.

## Pre-publication history

The pre-publication history for this paper can be accessed here:



## References

[B1] Wens J, Van Casteren V, Vermeire E, Van Royen P, Pas L, Denekens J (2001). Diagnosis and treatment of type 2 diabetes in three Belgian regions. Registration via a network of sentinel general practices. Eur J Epidemiol.

[B2] American Diabetes Association (2003). Screening for diabetes. Diabetes Care.

[B3] UK Prospective Diabetes Study Group (1998). Intensive blood glucose control with sulphonylureas or insulin compared with conventional treatment and risk of complications in patients with type 2 diabetes (UKPDS 33). Lancet.

[B4] UK Prospective Diabetes Study Group (1998). Effect of intensive blood glucose control with metformin on complications in overweight patients with type 2 diabetes. (UKPDS 34). Lancet.

[B5] UK Prospective Diabetes Study Group (1998). Tight blood pressure control and risk of macrovascular and microvascular complications in type 2 diabetes (UKPDS 38). BMJ.

[B6] UK Prospective Diabetes Study Group (1998). Efficacy of atenolol and captopril in reducing risk of both macrovascular and microvascular complications in type 2 diabetes (UKPDS 39). BMJ.

[B7] Anon (1997). Working Party from Complinace to concordance. Achieving goals in medicine taking. London, Royal Pharmaceutical Society of Great Britain.

[B8] Morris LS, Schulz RM (1992). Patient compliance: an overview. J Clin Pharm Ther.

[B9] Sackett DL, Snow JC, Haynes RB, Taylor DW, Sackett DL (1979). The magnitude of compliance and non-compliance. Compliance in Health Care.

[B10] Griffith S (1990). A review of the factors associated with compliance and the taking of prescribed medicines. British Journal of General Practice.

[B11] Roberson MHB (1992). The meaning of compliance: patient perspectives. Qualitative Health Research.

[B12] Alogna M (1980). Perceptions of severity of disease and health locus of control in compliant and non compliant diabetes patients. Diabetes Care.

[B13] Boom CAB, Hart LK (1980). The relationship between the health belief model and compliance of persons with diabetes mellitus. Diabetes Care.

[B14] Cahill J (1996). Patient participation: a concept analysis. J Advanced nursing.

[B15] Bissell P, May CR, Noyce PR (2004). From compliance to concordance: barriers to accomplishing a re-framed model of health care interactions. Social Science & Medicine.

[B16] Little P, Everitt H, Williamson I (2001). Preferences of patients for patient centred approach to consultation in primary care: observational study. BMJ.

[B17] Lagro-Janssen ALM, Van der Werf G Th (1999). Hoe spreken huisartsen en hun patiënten over ziekte? Een zoektocht in de medische literatuur naar het ziektebegrip van huisartsen en patiënten. Huisarts en Wetenschap.

[B18] Anderson RM, Robins LS (1998). How do we know? Reflections on qualitative research in diabetes. Diabetes Care.

[B19] Murri R, Antinori A, Ammassari A, Nappa S, Orofino G, Abrescia N, Mussini C, D'Arminio Monforte A, Wu AW, AdICoNA Study Group (2002). Physician estimates of adherence and the patient-physician relationship as a setting to improve adherence to antiretroviral therapy. J Acquir Immune Defic Syndr.

[B20] Hunt LM, Arar NH (2001). An analytical framework for contrasting patient and provider views of the process of chronic disease management. Med Anthropol Q.

[B21] Trestle JA (1988). Medical compliance as an ideology. Soc Sci Med.

[B22] Morgan DL, Krueger RA (1997). "Focus Group Kit" parts 1 to 6.

[B23] Mays N, Pope C (1995). Qualitative Research. Rigour and qualitative research. BMJ.

[B24] Murphy E, Dingwall R, Greatbatch D, Parker S, Watson P (1998). Qualitative research methods in health technology assessment: a review of the literature. Health Technology Assessment.

[B25] Vermeire E, Van Royen P, Coenen S, Wens J, Denekens (2003). The adherence of type 2 diabetes patients to their therapeutic regimens: patients' perspective. A qualitative study. Pract Diabetes Int.

[B26] Vermeire E, Hearnshaw H, Van Royen P, Denkens J (2001). Patient adherence to treatment: three decades of research. A comprehensive review. J Clin Pharm Ther.

[B27] Krueger RA (1988). Focus groups: a practical guide for applied research.

[B28] Morgan DL, Stewart M, Tudiver F, Bass MJ, Dunn EV, Norton PG (1992). Designing focus group research. Tools for primary care research.

[B29] Cramer JA (2004). A systematic review of adherence with medications for diabetes. Diabetes Care.

[B30] Burke LE, Fair J (2003). Promoting prevention: skill sets and attributes of health care providers who deliver behavioral interventions. Journal of Cardiovascular Nursing.

[B31] Botelho RJ, Novak S (1993). Dealing with substance misuse, abuse, and dependency. Prim Care.

[B32] Heszen-Klemens I (1987). Patients' non-compliance and how doctors manage this. Soc Sci Med.

[B33] Griffin S, Kinmonth AL (2003). Systems for routine surveillance for people with diabetes mellitus (Cochrane Review). The Cochrane Library.

[B34] Renders CM, Valk GD, Griffin S, Wagner EH, Eijk JThM van, Assendelft WJJ (2003). Interventions to improve the management of diabetes mellitus in primary care, outpatient and community settings. (Cochrane Review). The Cochrane Library.

[B35] Van den Arend IJ, Stolk RP, Krans HM, Grobbee DE, Schrijvers AJ (2000). Management of type 2 diabetes: a challenge for patient and physician. [Review]. Patient Education & Counseling.

[B36] Alazri MH, Neal RD (2003). The association between satisfaction with services provided in primary care and outcomes in Type 2 diabetes mellitus. Diabetic Medicine.

[B37] Mead N, Bower P, Hann M (2002). The impact of general practitioners' patient-centredness on patients' post-consultation satisfaction and enablement. Social Science & Medicine.

[B38] Lewin SA, Skea ZC, Entwistle V, Zwarenstein M, Dick J (2004). Interventions for providers to promote a patient-centred approach in clinical consultations. [Systematic Review]. Cochrane Consumers & Communication Group Cochrane Database of Systematic Reviews.

[B39] Kinmonth AL, Woodcock A, Griffin S, Spiegal N, Campbell MJ (1998). Randomised controlled trial of patient centred care of diabetes in general practice: impact on current wellbeing and future disease risk. BMJ.

[B40] Heisler M, Vijan S, Anderson RM, Ubel PA, Bernstein SJ, Hofer TP (2003). When do patients and their physicians agree on diabetes treatment goals and strategies, and what differences does it make?. Journal of General Internal Medicine.

[B41] Demmer C (2003). Relationship with health care provider and adherence to HIV medications. Psychological reports.

[B42] Demyttenaere K (2003). Risk factors and predictors of compliance in depression. Review. European Neuropsychopharmacology.

[B43] Brown JB, Harris SB, Webster-Bogaert S, Wetmore S, Faulds C, Stewart M (2002). The role of patient, physician and systemic factors in the management of type 2 diabetes mellitus. Family Practice.

[B44] Pope C, Van Royen P, Baker R (2002). Qualitative methods in research on healthcare quality. Qual Saf Health Care.

[B45] Health Survey Interview. http://www.iph.fgov.be/epidemio/epien/index4.htm.

[B46] Krupat E, Stein T, Selby JV, Yeager CM, Schmittdiel J (2002). Choice of a primary care physician and its relationship to adherence among patients with diabetes. Am J Manag Care.

[B47] Donovan JL (1995). Patient decision making. The missing ingredient in complex research. Int J Technol Assess Health Care.

